# A Study for Tooth Bleaching via Carbamide Peroxide-Loaded Hollow Calcium Phosphate Spheres

**DOI:** 10.3390/dj5010003

**Published:** 2016-12-26

**Authors:** Tao Qin, Torbjörn Mellgren, Steven Jefferies, Wei Xia, Håkan Engqvist

**Affiliations:** 1 Division for Applied Material, Department of Engineering Science, Uppsala University, Uppsala 75121, Sweden; tao.qin@angstrom.uu.se (T.Q.); torbjorn.mellgren@angstrom.uu.se (T.M.); wei.xia@angstrom.uu.se (W.X.); 2 Kornberg School of Dentistry, Department of Restorative Dentistry, Temple University, Philadelphia, PA 19140, USA; srj0573@temple.edu

**Keywords:** calcium phosphate, spheres, tooth bleaching, remineralization

## Abstract

The objective of this study was to investigate if a prolonged bleaching effect of carbamide peroxide-loaded hollow calcium phosphate spheres (HCPS) can be achieved. HCPS was synthesized via a hydrothermal reaction method. Carbamide peroxide (CP) was-loaded into HCPS by mixing with distilled water as solvent. We developed two bleaching gels containing CP-loaded HCPS: one gel with low HP concentration as at-home bleaching gel, and one with high HP concentration as in-office gel. Their bleaching effects on stained human permanent posterior teeth were investigated by measuring the color difference before and after bleaching. The effect of gels on rhodamine B degradation was also studied. To investigate the potential effect of remineralization of using HCPS, bleached teeth were soaked in phosphate buffer solution (PBS) containing calcium and magnesium ions. Both bleaching gels had a prolonged whitening effect, and showed a strong ability to degrade rhodamine B. After soaking in PBS for 3 days, remineralization was observed at the sites where HCPS attached to the teeth surface. CP-loaded HCPS could prolong the HP release behavior and improve the bleaching effect. HCPS was effective in increasing the whitening effect of carbamide peroxide and improving remineralization after bleaching process.

## 1. Introduction

Applying hydrogen peroxide or carbamide peroxide are established methods for teeth bleaching [1,2,3,4,5]. There are two established methods for vital tooth bleaching: in-office and at-home bleaching. Respectively, 35% hydrogen peroxide for in-office or 10%–20% carbamide peroxide (which equals 3.5%–6.5% hydrogen peroxide) or 3%–6% hydrogen peroxide for at-home bleaching [6].

However, the potential side effects should not be ignored. 80% of patients bleaching their teeth showed negative effects according to a report including input from more than 7000 dentists [1]. Tooth sensitivity, rebound of stain, enamel surface change, and soft tissue irritation are generally regarded. Among them, tooth sensitivity is the most commonly reported side effect [7].

Dentin sensitivity is generally referring to patients experiencing a sharp pain due to exposed dentin tubules [8]. The exposure of tubules may be initialized by the use of bleaching products, which can result in surface changes and increased roughness. Surface changes and roughness can be caused by carbamide peroxide with or without the addition of carbopol and glycerin [2]. The use of 10% carbamide peroxide has been found to cause mild, reversible histological changes in some patients [9]. To minimize tooth sensitivity, lowering the concentration of peroxide in the paste has been recommended [10]. 

Some strategies have been used to reduce tooth sensitivity caused by peroxide bleaching [5,11,12]. For example, Prospec MI paste (GC America) containing casein phosphopeptide-amorphous calcium phosphate (CPP-ACP) have been found effective in reducing sensitivity [11]. The paste is recommended to be applied immediately after bleaching. In addition, some specific agents can be mixed into bleaching paste to obtain less sensitivity (e.g., 5% potassium nitrate as a desensitizer [12] and amorphous calcium phosphate (ACP)-containing bleaching gel [5]). Tooth remineralization is another good strategy to relieve the sensitivity. Previous studies have proven that calcium phosphate can improve tooth remineralization [13,14,15].

This paper provides new insights regarding sustained release of peroxide via hollow calcium phosphate spheres (HCPS). We investigated the bleaching effect of at-home gel and in-office gel, all containing peroxide-loaded HCPS. HCPS which adhere to enamel surface are believed to prolong bleaching effect and improve remineralization. The remineralization effect after bleaching was studied via soaking of bleached tooth slabs in phosphate buffer solution (PBS) and evaluated by SEM. HCPS could be an alternative hydrogen peroxide carrier and remineralization agent in bleaching gels. 

## 2. Materials and Methods

### 2.1. Materials

Chemicals were purchased from Sigma-Aldrich (Sweden). HCPS was synthesized as described below.

HCPS was prepared based on a previously published method [16]. In summary, strontium nitrate (0.6 mM) was added in as-prepared phosphate buffer solutions with calcium and magnesium ions. The above solution was then sealed in a glass bottle, and kept in an oven at 100 °C for 24 h. The obtained precipitation was filtered, washed with ethanol, and dried at 60 °C for further usage. The morphology was evaluated by field emission scanning electron microscopy (FESEM, LEO 1550, Zeiss, Oberkochen, Germany).

### 2.2. Carbamide Peroxide-Loaded HCPS and Gels Preparation

Carbamide Peroxide (CP)-loaded HCPS were prepared by following steps. HCPS (0.5 g), 9.5 mL water, and 2.475 g carbamide peroxide were mixed at room temperature and stirred in dark condition for 2 h. The suspension was then filtered, and the mixture was dried in dark condition for 24 h in room temperature. 

Modified CP-loaded HCPS were prepared under low temperature and certain ratio of hydrogen peroxide and urea. The loading was done at 9 °C, in a water bath cooled by ice. Hydrogen peroxide and urea (ratio 1.2:1) was added to a water suspension with HCPS and stirred for 50 min. The loaded spheres were then separated from the suspension and dried in dark condition for 24 h at room temperature. 

The at-home gel and in-office gel were prepared according to Table 1. CP-loaded HCPS was only mixed with anhydrous glycerol to make a low concentration of at-home gel. Extra HP was added to CP-loaded HCPS to prepare in-office gel. The control gel was chairside tooth whitening agent, Nupro^®^ White Gold (36% Hydrogen Peroxide, Just4teeth Inc., Chatsworth, CA, USA) gel.

### 2.3. HP Release from Loaded HCPS

The release study was carried out by the following steps. CP-loaded HCPS (0.1 g) was put into 10 mL distilled water in a 20 mL glass bottle. Three bottles (containing three samples) were placed in static and dark condition at room temperature. 

At each sampling, 500 μL solution was extracted from the top clear part of the overall solution. Samplings were performed at certain time points: 10 min, 30 min, 60 min, 90 min, 150 min, and 300 min. The extracted solutions were then diluted before measurement. The measurement was carried out by hydrogen peroxide test kit (Hanna instruments, Romania). This method is called titrimetric method according to product instructions. Iodide ions can be oxidized by hydrogen peroxide into iodine, which is blue. The iodine can be reduced back by titrating with standard sodium thiosulfate solution to iodide ions, which is colorless. The concentration of HP can be calculated by the drops of standard sodium thiosulfate solution. 

### 2.4. Tooth Staining and Bleaching

Extracted human permanent posterior teeth free of lesions or obvious defects were collected under an Institutional Review Board (IRB) exempt protocol. Teeth were stored in sterile distilled water at 4 °C for up to 2 weeks prior to processing. The outer surface of each specimen was cleaned with a soft-bristle toothbrush and sterile distilled water. 

The in vitro tooth staining and stain assessment method was adapted and modified from a previous study [17]. The sectioned human teeth were stained internally with a concentrated tea solution for a sufficient time at 4 °C until a stable external shade of Vita C4 was achieved. After completion of internal staining with the tea solution, the exposed dentin surfaces were sealed with clear nail polish. Baseline and treatment L*a*b* were assessed using a Vita Easyshade^®^ linical spectrophotometer. 

Both at-home gel and in-office gel were applied on the teeth surface, and kept in a moist environment under 37 °C for 45 min. This was the bleaching process, in which the teeth were exposed to peroxide. Then, the gel was rinsed using water. Vita Easyshade was then used to measure the whiteness of teeth again. 

The bleaching gels were washed away from bleached teeth in order to investigate the prolonged bleaching effect of HCPS. The bleached teeth were further kept in a moist environment. The teeth bleached by in-office gel were kept in a moist environment for 15 min and 24 h. The teeth bleached by at-home gel were kept in a moist environment for 2 h. Then, the whiteness of all teeth was also measured by Vita Easyshade. 

### 2.5. Rhodamine Degradation

Once the bleaching gels were placed into rhodamine B solution, hydrogen peroxide was released, which could react with rhodamine B at room temperature. Three samples of at-home and in-office bleaching gels (0.5 g) were placed in three 10 mL 0.5 M rhodamine solution, respectively. During sampling, a 500 μL aliquot was extracted from each bottle. Aliquots were extracted after 10, 30, 60, 90, 150, and 210 min.

The extracted solutions were then diluted before measurement. The measurement was carried out by a UV-1800 spectrophotometer (Shimadzu, Kyoto, Japan).

### 2.6. Color Evaluation

The images of teeth were captured by a digital camera under the same conditions.

The color values and color difference were evaluated according to CIE L*a*b color coordinate system. L, a, b values represent degree of lightness, greenness or redness and blueness or yellowness, respectively. L, a, b values were measured by Vita Easyshade (Vident, Brea, California).

The overall color difference ∆E was determined by Equation (1).

(1) $$\Delta E=\sqrt{\Delta L^{2}+\Delta a^{2}+\Delta b^{2}}$$

∆L, ∆a, ∆b are the changes in L, a, b, respectively.

### 2.7. Remineralization

After bleaching, the teeth were soaked in 2 mL PBS, which was kept in an oven at 37 °C. Four samples were kept for 0 and 3 days, respectively. The morphology of the teeth surfaces were evaluated by SEM. The samples were sputtered with Au/Pd before analysis.

## 3. Results

### 3.1. HCPS

**Figure 1:**
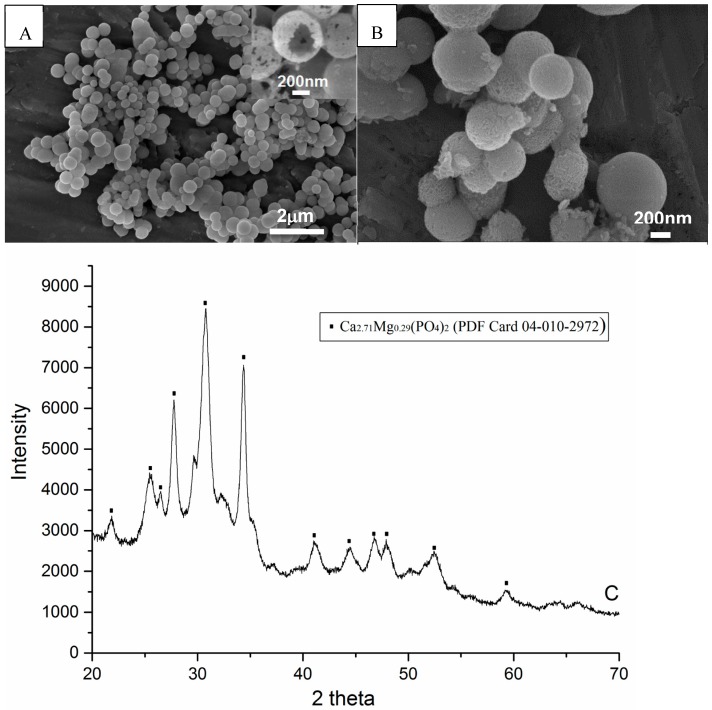
SEM images and XRD pattern of hollow calcium phosphate spheres synthesized by hydrothermal method. (**A**) Spheres dried at 60 °C for 2 h after synthesis; (**B**) Spheres loaded with hydrogen peroxide for 2 h; (**C**) X-ray diffraction pattern of (A), which indicates that the composition of spheres is magnesium substituted calcium phosphate.

SEM analyses showed that the spherical particles were hollow (see Figure 1A). There was no significant morphological difference after 2 h mixing with hydrogen peroxide, compared to the original spheres (see Figure 1B). The XRD pattern of spheres indicated the phase to be mainly magnesium substituted calcium phosphate (see Figure 1C).

### 3.2. Hydrogen Peroxide Release

**Figure 2:**
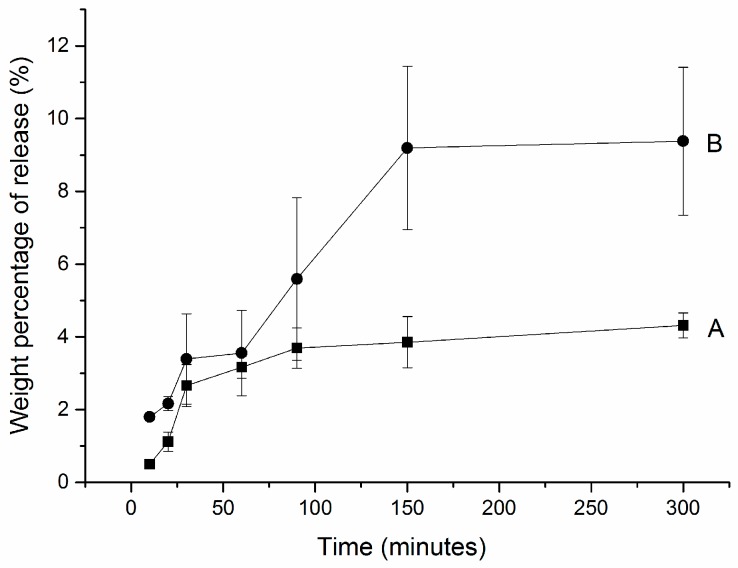
Hydrogen peroxide release profiles from (**A**) CP-loaded HCPS and (**B**) modified CP-loaded HCPS. (**A**) 0.5 g spheres, 2.475 g CP, and 9.5 mL water were mixed with stirring for 2 h at room temperature; (**B**) 0.5 g spheres, 2.475 g CP, 0.5 g 35% HP (total mole ratio of HP: urea = 1.2:1), and 9.5 mL water mixed with stirring for 2 h at 9 °C.

During the first 30 min, there was a burst release of hydrogen peroxide from CP-loaded HCPS (the sample was loaded under room temperature)—see Figure 2A. From 30 min to 100 min, linear release was observed. At 300 min, total release amount was 4.31 mg (±0.33 mg); 4.31% hydrogen peroxide was released from CP-loaded HCPS.

The modified CP-loaded HCPS—which was loaded at 9 °C with a mole ratio of HP and urea of 1.2:1—showed a similar release profile as samples loaded in room temperature (see Figure 2B). During the first 30 min, there was also a burst release of hydrogen peroxide. At the end, however, much more (8%) of hydrogen peroxide was released from the modified CP-loaded HCPS.

### 3.3. Tooth Bleaching

**Figure 3:**
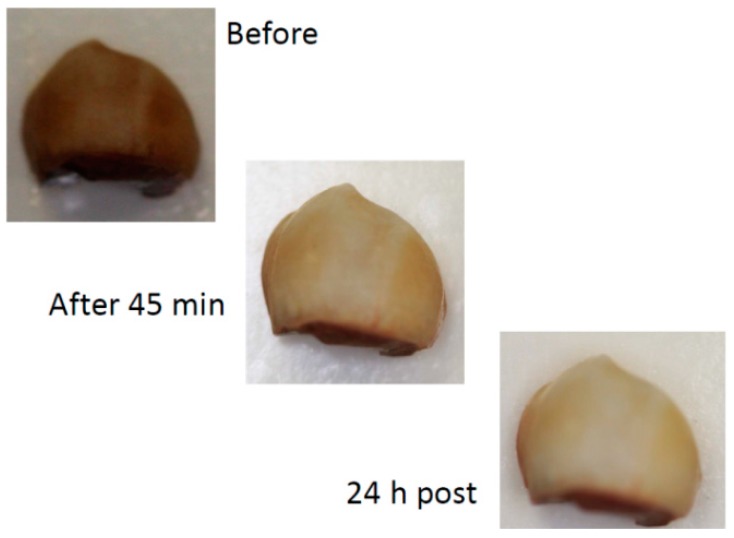
Example of the effect of 45 min bleaching in-office and 24 h after bleaching procedure and wet storage (digital camera).

**Figure 4:**
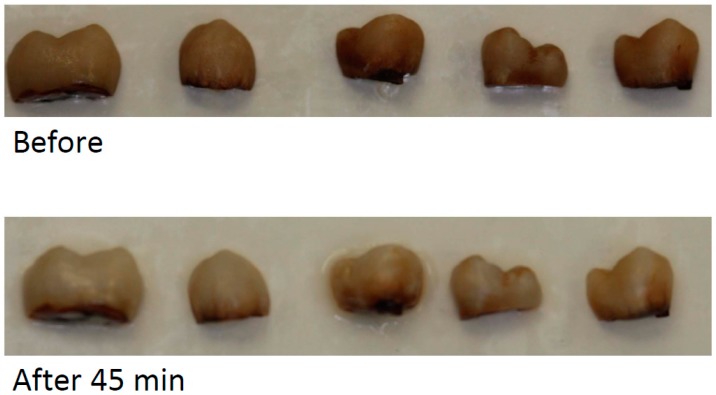
Examples of bleaching effect after 45 min bleaching by at-home bleaching gel.

A marked whitening effect was observed after 45 min bleaching by in-office gel (see Figure 3). For the at-home gel, the whitening was less after 45 min bleaching (Figure 4). 

Table 2 shows the ∆L, ∆a, ∆b, and ∆E of teeth bleaching by control, at-home gel, and in-office gel. After 45 min bleaching, mean ∆E of control gel, in-office gel, and at-home gel were 24.83, 16.56, and 11.89, respectively. Interestingly, mean ∆E became greater after the gel was washed away. For the in-office gel, it increased to 16.68 and 20.87 after 15 min and 24 h. For the at-home gel, it increased to 12.68 after 2 h.

Here are some explanations for the nomenclature in the table. For example, 45 min bleaching via in-office gel: tooth was bleached for 45 min with in-office gel. Fifteen minutes after washing via in-office gel, in-office gel was washed away from the tooth, which was kept in a moist environment for 15 min at 37 °C. 

### 3.4. Rhodamine B Degradation

**Figure 5:**
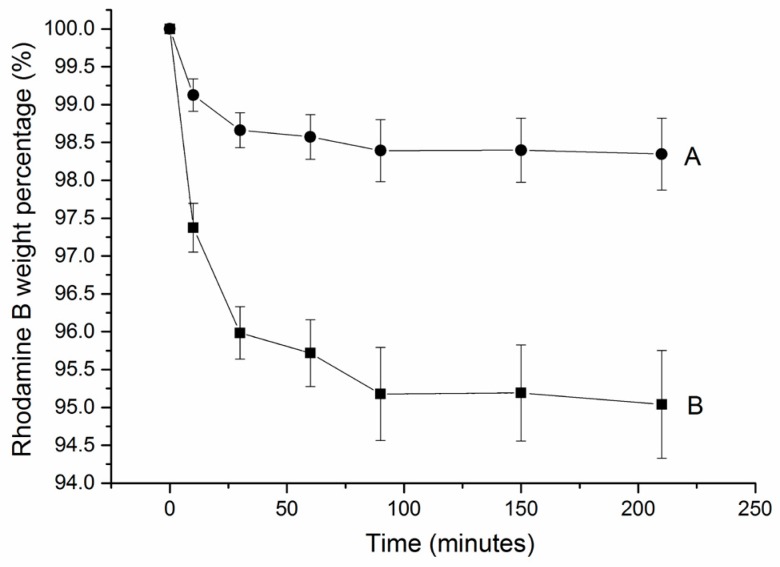
Rhodamine B degradation profile (average and standard deviation) via (**A**) at-home gel and (**B**) in-office gel. Each 0.5 g gel was placed in 10 mL 0.5 M rhodamine B solution for 250 min. For each gel, n = 3.

Rhodamine B (1 wt%) was degraded via at-home gel in the first 25 min (see Figure 5A). In the following 65 min, only approximately 0.5 wt% was observed. For the in-office gel, 4 wt% of rhodamine B was degraded in the first 25 min (see Figure 5B). In the following 65 min, only approximately 1 wt% was observed. The ability of the bleaching gels to degrade rhodamine B was dramatically decreased in 90 min. 

### 3.5. Tooth Remineralization

**Figure 6:**
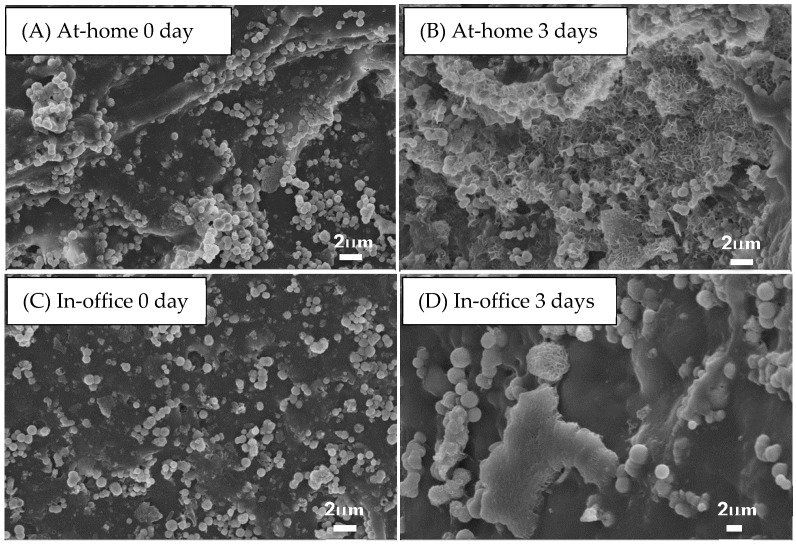
Morphology of bleached teeth surface after being rinsed in 2 mL phosphate buffer solution (PBS) at 37 °C for 0 days (**A**,**C**) and 3 days (**B**,**D**). (**A**,**B**) and (**C**,**D**) were bleached by at-home gel and in-office gel, respectively.

After teeth were treated by at-home gel and in-office gel, HCPS were observed on the enamel surface via SEM (see Figure 6A,C). After soaking in PBS for 3 days, new apatite was observed at the sites where HCPS attached (see Figure 6B,D).

## 4. Discussion

The SEM images showed the hollow structure of spheres. The hollow structure of the spheres was also proved by transmission electron microscopy in a previous study [16].

The concentration of CP in CP-loaded HCPS was around 16%, but (as is well known) the instability of the peroxide makes it difficult to determine the exact amount. Hydrogen peroxide easily decomposes into water and oxygen. During loading and drying processes, alumina foil was used to keep samples dark in order to reduce the decomposition. In this paper, the release rate of hydrogen peroxide averaged 4% after 150 min. The release of HP from CP-loaded HCPS could last at least 50 min. Even if the concentration of the at-home bleaching gel was low, the prolonged release kept the bleaching effect for at least 1 h. 

Both at-home and in-office gels were effective in bleaching. The concentration of HP in the in-office gel was higher, compared to the at-home gel. Correspondingly, its bleaching effect was much more obvious. As mentioned in the introduction, the lower concentration, the less sensitivity caused. The high concentration of HP is a common drawback of bleaching products [2]. The most significant finding was the prolonged bleaching effect, which could change the traditional conception of bleaching. In traditional bleaching, the bleaching occurs during the gel posting on the enamel [17]. In this study, the bleaching also continued after the exposure, which was due to peroxide-loaded HCPS adhering on the enamel. 

The degradation process of rhodamine B was based on oxidation by hydrogen peroxide [18] (as for bleaching). Degradation of rhodamine B oxidized by HP was same as teeth bleaching process. The bleaching gels had an obvious effect to degrade the rhodamine B at first 25 min and sharply decreased effect in next 60 min. Therefore, the bleaching effect of bleaching gels could last approximately 90 min. Compared to the at-home gel, the in-office gel had much more obvious effect because in-office gel contained higher concentration of HP.

The results from the remineralization tests showed the spheres could improve apatite formation on the enamel surface, which was also proved by our previous study on dentin surface [19]. Calcium phosphate spheres acted as starting points of apatite nucleation, which improved teeth remineralization. The newly formed apatite potentially helps to maintain the surface structure after bleaching and reduce tooth sensitivity. 

Through our studies, hollow calcium phosphate spheres could be a very promising filler material in tooth bleaching. 

## 5. Conclusions

In this paper, we investigated HCPS’s loading capacity of carbamide peroxide and its release behavior. Two bleaching gels—both containing CP-loaded HCPS—were studied for bleaching effect. Adding HCPS into bleaching products did not affect the bleaching effect. Meanwhile, HCPS was effective in prolonging the bleaching effect and improving teeth remineralization. The tested bleaching gels containing HCPS could be an alternative of the presently used bleaching products.

## Figures and Tables

**Table 1:** The composition of at-home gel and in-office bleaching gel. CP: carbamide peroxide; HCPS: hollow calcium phosphate spheres.

(wt%)	CP-Loaded HCPS	Anhydrous Glycerol	Others	Estimated Maximal Whitening Agent Wt%
At-home gel	50%	50%	No	6% HP
In-office gel	50%		50% HP(35%)	20% HP

**Table 2:** Mean (± standard deviation) color changes after tooth bleaching via control gel (36% hydrogen peroxide), at-home gel, and in-office gel.

Bleaching	∆L	∆a	∆b	∆E
Control with 45 min bleaching	22.30	−10.90	−0.70	24.83
45 min bleaching via in-office gel	11.43 ± 3.32	−8.40 ± 2.34	−8.55 ± 2.91	16.56 ± 5.00
15 min after washing via in-office gel	14.58 ± 3.26	−7.07 ± 2.42	−3.97 ± 3.07	16.68 ± 5.09
24 h after washing via in-office gel	20.05 ± 3.63	−5.78 ± 2.39	0.20 ± 1.66	20.87 ± 4.65
45 min bleaching via at-home gel	9.08 ± 3.42	−5.74 ± 1.63	−5.10 ± 3.24	11.89 ± 4.48
2 h after washing via at-home gel	8.94 ± 2.81	−6.46 ± 1.72	−6.26 ± 3.01	12.68 ± 4.47
